# Multimodal imaging of bilateral diffuse uveal melanocytic proliferation associated with an iris mass lesion

**DOI:** 10.1186/s40942-016-0038-7

**Published:** 2016-05-16

**Authors:** Jonathan Naysan, Claudine E. Pang, Robert W. Klein, K. Bailey Freund

**Affiliations:** 1 The Vitreous Retina Macula Consultants of New York, 460 Park Avenue, Fifth Floor, New York, NY 10022 USA; 2 LuEsther T. Mertz Retinal Research Center, Manhattan Eye, Ear and Throat Hospital, New York, NY USA; 3 Department of Ophthalmology, New York University School of Medicine, New York, NY USA; 4 Department of Ophthalmology, North Shore - Long Island Jewish Health System, New York, NY USA

**Keywords:** Bilateral diffuse uveal melanocytic proliferation, Melanocytes, Malignancy, Ultra-widefield, Swept-source optical coherence tomography, Iris tumor

## Abstract

**Background:**

Bilateral diffuse uveal melanocytic proliferation (BDUMP) is a rare, paraneoplastic syndrome characterized by bilateral painless visual loss and proliferation of choroidal melanocytes in association with an underlying systemic malignancy. We report a case of bilateral diffuse uveal melanocytic proliferation associated with an underlying gynecological malignancy that also features the infrequent finding of an iris mass lesion, using multimodal imaging including ultra-widefield imaging, spectral domain and swept-source optical coherence tomography.

**Case presentation:**

A 59-year-old white female with a prior history of gynecological malignancy in remission presented with progressive bilateral visual loss over several weeks. The patient was noted to have a focal iris mass lesion in her right eye. Ultra-widefield color fundus photography showed a characteristic bilateral ‘giraffe pattern’ of pigmentary changes extending into the periphery as well as multiple discrete deeply pigmented lesions. Ultra-widefield autofluorescence was useful for visualizing the full extent of involvement. Indocyanine green angiography helped to demarcate the discrete pigmented choroidal lesions. Swept-source OCT clearly delineated the alternating zones of retinal pigment epithelium (RPE) thickening and RPE loss, as well as the prominent choroidal infiltration and thickening.

**Conclusions:**

BDUMP is an important diagnosis to consider in the presence of multiple discrete melanocytic choroidal lesions, diffuse choroidal thickening, characteristic RPE changes, iris mass lesions and exudative retinal detachment. Ultra-widefield imaging may demonstrate more extensive lesions than that detected on clinical examination or standard field imaging. Imaging with SS-OCT shows choroidal and RPE characteristics that correlate well with known histopathology of this entity.

## Background

Bilateral diffuse uveal melanocytic proliferation (BDUMP) is a rare, paraneoplastic syndrome characterized by bilateral painless, profound visual loss in association with an underlying systemic malignancy [1]. In 1966, Machemer et al. [2] first described this clinical syndrome, although it was not until 1982 that Barr et al. [3] coined the term BDUMP. In 1990, Gass et al. [4] described the five cardinal signs of BDUMP as: (1) multiple orange-red subretinal patches; (2) early hyperfluorescence on fluorescein angiography; (3) multiple elevated pigmented and non-pigmented uveal melanocytic tumors with diffuse choroidal thickening; (4) exudative retinal detachments; and (5) rapidly progressive cataracts.

Since then, about 50 cases have been described in the literature [5–32] and the primary malignancy typically involves the reproductive tract in women, and gastrointestinal or respiratory system in men. We report an unusual case of BDUMP in a patient with a history of ovarian carcinoma. This case also displayed the uncommon finding of an iris mass lesion, which has been rarely described in this entity. This case of BDUMP was evaluated with multimodal imaging techniques that included ultra-widefield color and autofluorescence (AF); indocyanine green angiography (ICGA); and spectral-domain (SD) and swept-source (SS) optical coherence tomography (OCT).

## Case presentation

**Fig. 1:**
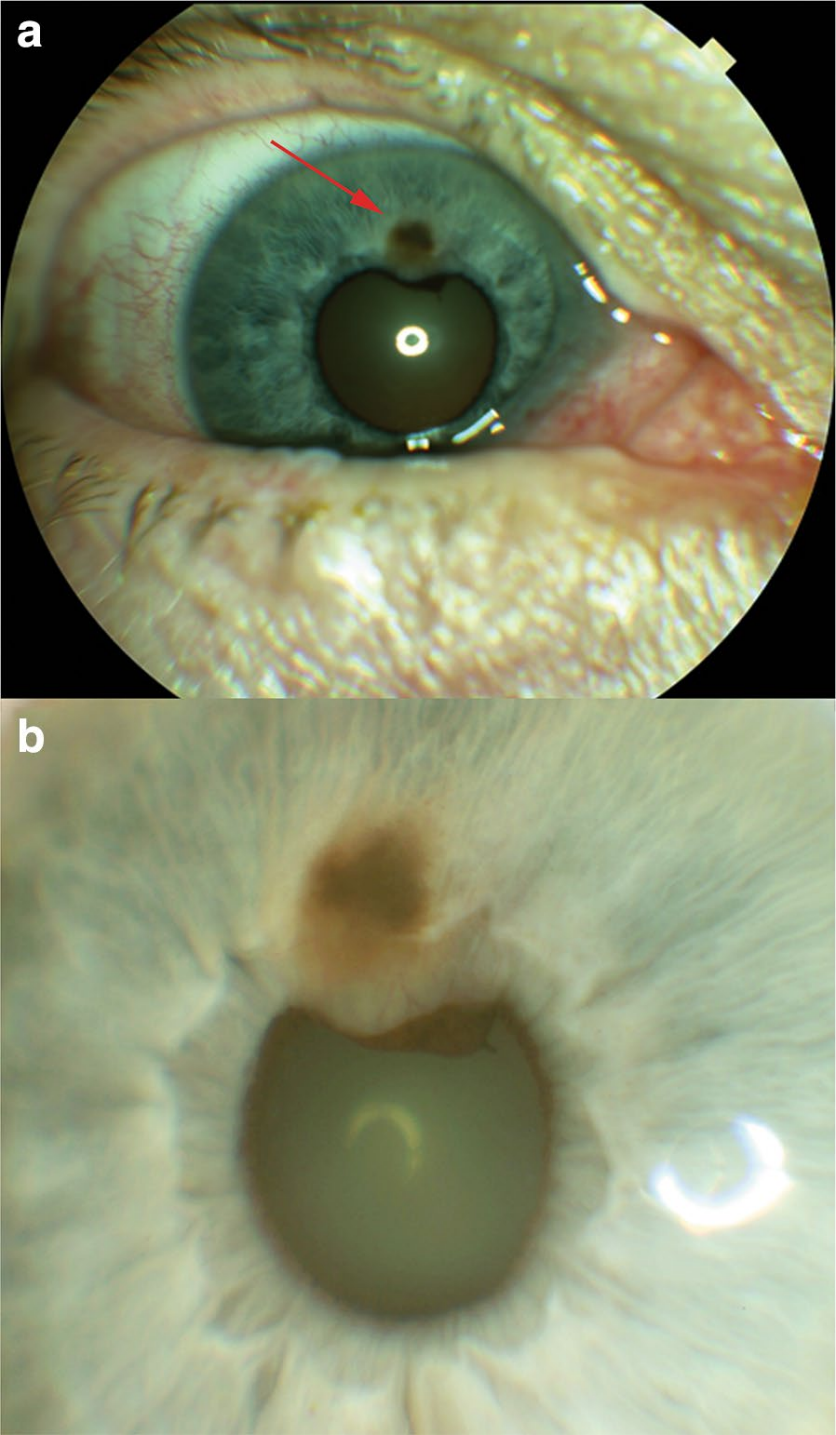
Color photograph of patient’s right eye showing a superior iris melanocytic lesion (**a**). Higher magnification view of the iris lesion displaying the pigmented mass with some associated vascularity (**b**)

**Fig. 2:**
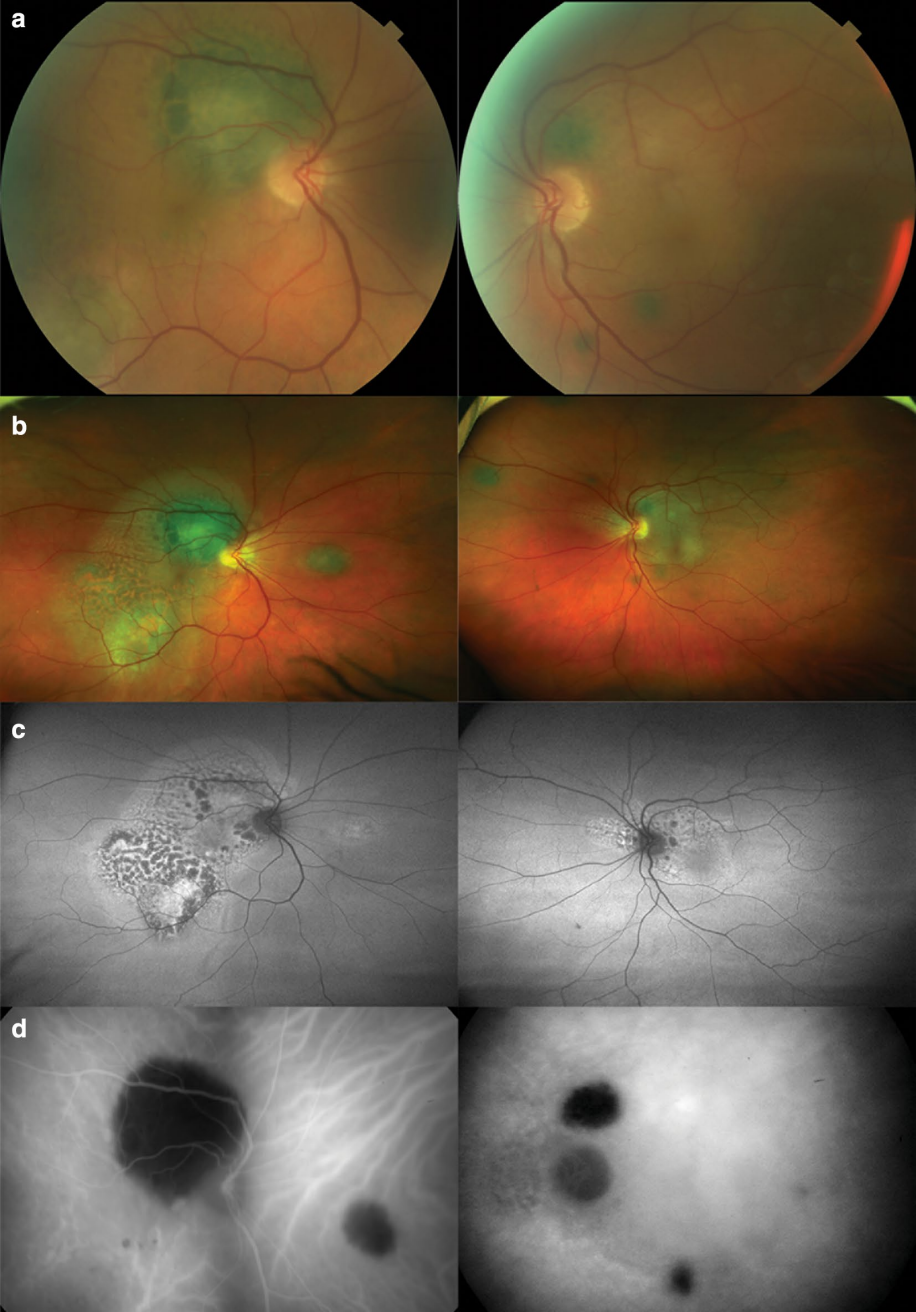
Standard flood-illuminated and Ultra-wide field (UWF) color photographs show multiple scattered melanocytic lesions throughout the fundus associated with pigmentary changes extending from the posterior pole (**a**, **b**). UWF AF shows round-like patches of hypo-AF alternating with intervening areas of hyper-AF in the posterior pole (**c**). ICG angiography shows these scattered melanocytic lesions to be hypofluorescent (**d**)

A 59-year-old white female presented with complaints of decreasing vision and photophobia over the prior month. Her medical history was notable for hypertension and chemotherapy for ovarian carcinoma 5 years prior to presentation. On examination, her best-corrected visual acuity was 20/40 OD and 20/80 OS. The anterior segment examination was remarkable for a pigmented, vascularized iris mass lesion at the superior pupillary border in her right eye (Fig. 1). Her intraocular pressures were normal. Funduscopic examination and color fundus photography (Topcon TRC-50XF; Topcon Medical Systems, Oakland, NJ) showed bilateral exudative macular detachments with multiple scattered darkly pigmented choroidal lesions (Fig. 2a). The extent of the pathology was better seen with ultra-widefield color images (Optos 200Tx; Optos, Scotland, United Kingdom) (Fig. 2b). RPE changes forming a ‘giraffe-pattern’ were noted in the posterior pole of both eyes. Ultra-widefield autofluorescence (AF) imaging (Optos 200Tx; Optos, Scotland, United Kingdom) highlighted and documented the full extent of this pattern with patches of hypo-AF alternating with intervening areas of hyper-AF (Fig. 2c). Fundus camera based ICGA (Topcon TRC-50XF; Topcon Medical Systems, Oakland, NJ) showed the scattered darkly pigmented choroidal lesions as well-circumscribed hypofluorescent areas (Fig. 2d).

**Fig. 3:**
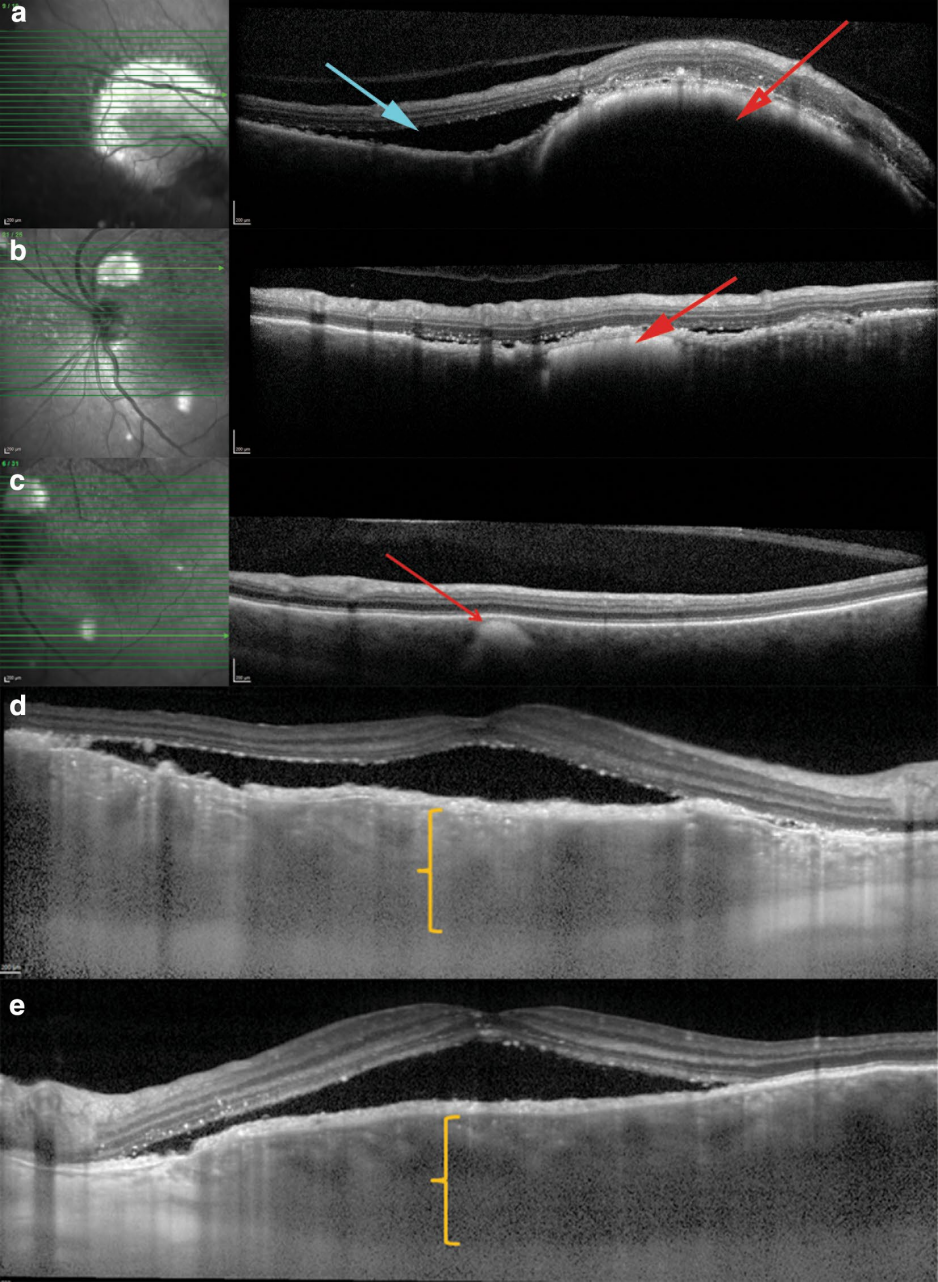
Spectral-domain optical coherence tomography (SD-OCT) with matching near-infrared reflectance (nIR) localizes these discrete lesions to the choroid with compression of the choriocapillaris (*red arrow*) and associated subretinal fluid (*blue arrow*). The choroidal lesions are intensely hyper-reflective on nIR images (**a**–**c**). EDI-OCT shows markedly increased choroidal thickness with overlying subretinal fluid of the right (**d**) and left eyes (**e**)

**Fig. 4:**
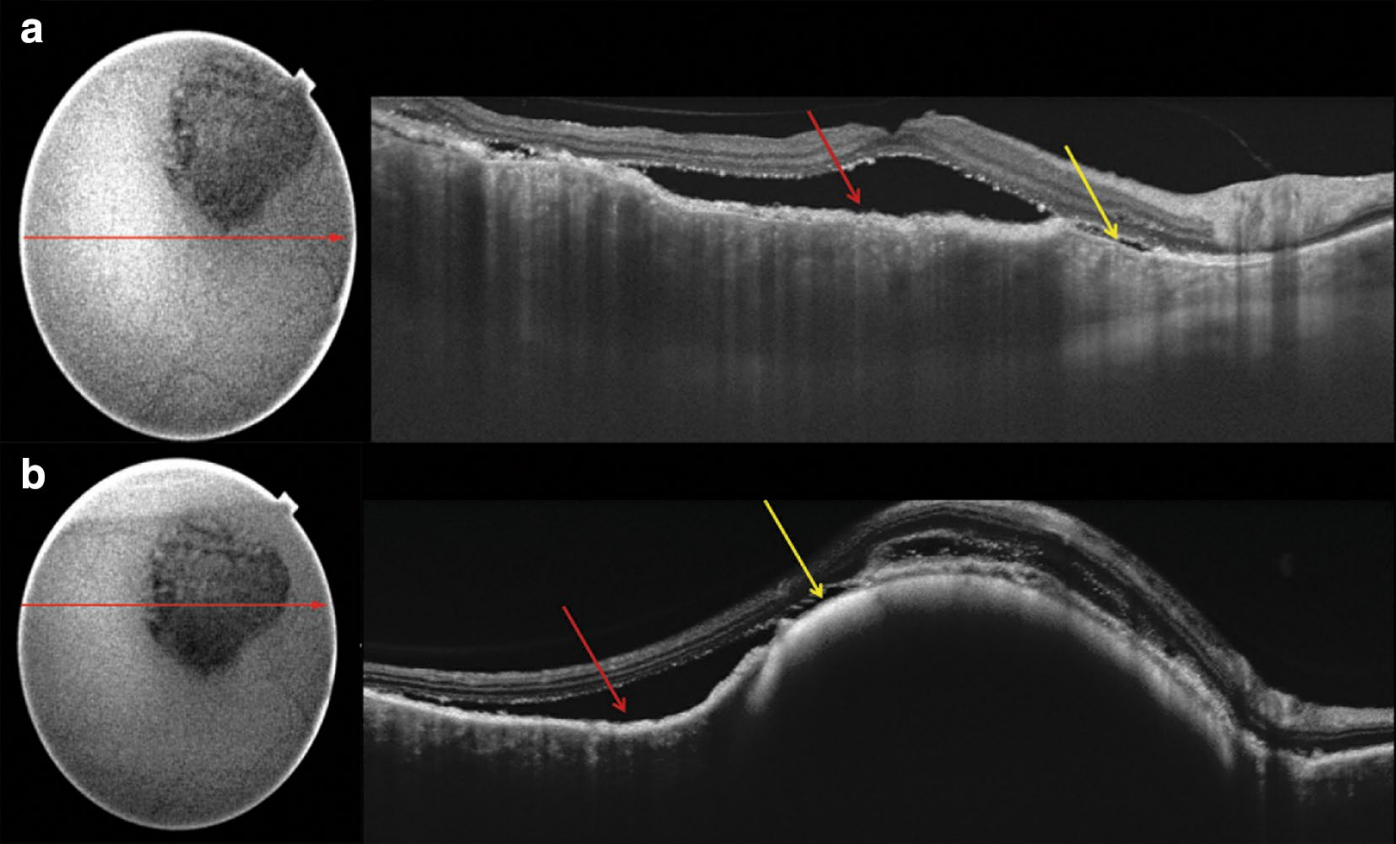
Swept-source optical coherence tomography (SS-OCT) through the fovea (**a**) and through a superior large melanocytic lesion (**b**) of the right eye highlighting the increased choroidal thickness, subretinal fluid and the alternating RPE thickening (*red arrow*) and RPE loss (*yellow arrow*) that corresponds to the appearance of the alternating pattern seen on the UWF AF

SD-OCT (Heidelberg Spectralis: Heidelberg Engineering, Heidelberg, Germany) showed areas of subretinal fluid and discrete hyper-reflective choroidal lesions that appear to be compressing the adjacent choriocapillaris. The choroidal lesions were highly reflective on near-infrared reflectance (nIR) (Fig. 3a–c). Enhanced depth imaging OCT (EDI-OCT) performed with the Heidelberg Spectralis showed diffuse choroidal thickening beneath the exudative macular detachments (Fig. 3d, e). Swept-source OCT (DRI OCT-1; Topcon Medical Systems, Oakland, NJ) of the right eye showed alternating RPE thickening and RPE loss that corresponded to the appearance of the “giraffe-pattern” seen on the AF (Fig. 4).

The patient was referred back to her oncologist for further evaluation of a presumed recurrence of her ovarian carcinoma.

## Conclusions

We present a case of BDUMP using multimodal imaging including the use of ultra-widefield imaging, ICG angiography and SS-OCT that helped to show the full extent of the posterior segment disease. Ultra-widefield color fundus photography allowed greater visualization of pigmentary changes and multiple pigmented lesions in the periphery, while ultra-widefield AF accentuated the characteristic ‘giraffe-pattern’ fundus changes which were more difficult to appreciate on clinical examination. EDI-OCT and SS-OCT readily showed choroidal thickening and alternating zones of RPE thickening and RPE loss. These findings correlate well with the known histopathology of BDUMP. The choroid is diffusely thickened with uveal melanocytic cells including nevoid, spindle cells, and epithelioid cells. There are also aggregates of focal infiltration of pigmented melanocytes in the choroid with overlying areas of RPE destruction alternating with areas of RPE hypertrophy [1].

Of the approximately 50 cases of BDUMP reported in literature [1–33], only 6 cases have reported the association with iris tumors. [3, 20, 34–37] Originally described by Gass et al. [4] iris involvement is infrequently seen or documented. We highlight this uncommon occurrence to create awareness that BDUMP can lead to infiltration of the uveal tract including the iris and ciliary body, which may further lead to angle-closure glaucoma and cataracts.

Various treatments for BDUMP, including ocular radiation [1, 3, 19, 32], subretinal fluid drainage [30] and corticosteroids [15, 16, 24, 35] have been tried without success. Recently, plasmapheresis has been shown to improve visual acuity in few case reports. [1, 11] The rationale is to decrease the circulating autoantibodies, [1, 11, 24] shown to be in the IgG fragment termed cultured melanocyte elongation and proliferation (CMEP) factor, [38] which stimulates cutaneous and uveal melanocyte cell proliferation. However, success with this treatment has been limited. The overall prognosis for BDUMP is poor with the majority of patients passing within 3 years [39]. Treatment of the underlying systemic malignancy is crucial.

In summary, we show the benefit of multimodal imaging in BDUMP to demonstrate the full extent of the disease. We highlight the occurrence of iris lesions in this condition and the difficulties with treatment options.

## Abbreviations

BDUMP: bilateral diffuse uveal melanocytic proliferation
OCT: optical coherence tomography
SS: swept source
SD: spectral domain
AF: autofluorescence
ICGA: indocyanine green angiography
RPE: retinal pigment epithelium
EDI: enhanced depth imaging
UWF: ultra-wide field
